# Long-Term Sex-Specific Effects of Cadmium Exposure on Osteoporosis and Bone Density: A 10-Year Community-Based Cohort Study

**DOI:** 10.3390/jcm11102899

**Published:** 2022-05-20

**Authors:** Seung Min Chung

**Affiliations:** Division of Endocrinology and Metabolism, Department of Internal Medicine, Yeungnam University College of Medicine, Daegu 42415, Korea; smchung@ynu.ac.kr; Tel.: +82-53-620-4292; Fax: +82-53-654-8386

**Keywords:** cadmium, endocrine-disrupting chemicals, osteoporosis, quantitative ultrasound

## Abstract

This study explored the long-term effects of cadmium (Cd) exposure on osteoporosis incidence and bone mineral density (BMD). This retrospective cohort study included men aged ≥50 years and post-menopausal women from the 2001–2002 Korea Genome and Epidemiology Study. Participants previously diagnosed with osteoporosis were excluded. Blood Cd concentrations were measured and categorized as <0.5, 0.5–1.0, and >1.0 μg/L. BMD was measured using quantitative ultrasound. Osteoporosis was diagnosed when the T-score was ≤−2.5. Confounders that affect exposure and outcome were controlled. Osteoporosis incidence and differences in BMD (ΔBMD) were assessed until 2012. The osteoporosis incidence among 243 participants who were followed up for an average of 6.3 years was 22.2%. In all the participants, a dose–response relationship was observed between blood Cd and incident osteoporosis and ΔBMD (both *p*-for-trend < 0.01). After adjusting for age, sex, smoking, physical activity, body mass index, creatinine, and baseline BMD, a blood Cd concentration of >1.0 μg/L was an independent risk factor for incident osteoporosis and decrements in ΔBMD. In women, blood Cd concentrations of >0.5 μg/L increased the risk for osteoporosis. Exposure to Cd prospectively increases the risk for osteoporosis and decrements of ΔBMD, particularly in women, even in lower doses of Cd.

## 1. Introduction

Cadmium (Cd) is a well-known heavy metal recognized as an endocrine-disrupting chemical. Cd bioaccumulates in the human body through cigarette smoking and consumption of contaminated food and drinking water [1]. A few studies have reported that Cd exposure is associated with thyroid dysfunction [2], hepatic fibrosis [3], obesity, and diabetes [4].

The risk for fracture and osteoporosis is increased by advanced age, early menopause, smoking, excessive alcohol intake, low body weight, prior history of fracture without major trauma, or particular disease and treatments [5]. Additionally, Cd toxicosis due to industrial poisoning can induce osteoporosis, which is referred to as “Itai-itai” disease [6]. The association of Cd exposure with osteoporosis in the general population was also revealed through a small number of cross-sectional studies [7]. In three European countries, up to 23% of osteoporosis cases may be attributable to Cd exposure, and absolute costs associated with the burden of osteoporosis-related fractures attributable to Cd ranged between 0.12 and 2.6 billion EUR [8]. Despite this influence, the prospective and sex-specific impact of Cd exposure on incident osteoporosis and changes in bone mineral density (BMD) has not been corroborated.

For this purpose, we conducted a 10-year community-based cohort study to evaluate Cd exposure based on blood Cd levels and its effect on incident osteoporosis and changes in BMD.

## 2. Materials and Methods

### 2.1. Subjects and Methods

This retrospective cohort study used data obtained from the Korea Genome and Epidemiology Study (KoGES)-Ansan and Ansung cohorts [9]. The KoGES has been repeated every 2 years since the baseline survey in 2001–2002, which included 10,030 participants. We initially selected 499 participants (247 men and 252 women aged ≥40 years) with data on blood heavy metal concentration in the baseline survey. BMD testing was repeatedly performed until the sixth visit (2011–2012). The exclusion criteria were: (1) men aged <50 years (*n* = 89); (2) pre-menopausal women (*n* = 87); (3) participants previously diagnosed with osteoporosis and currently undergoing treatment (*n* = 26); and (4) participants whose BMD were examined only once between 2001 and 2012 (*n* = 54). A total of 243 participants were enrolled in the final analysis.

### 2.2. Assessment of Cadmium Exposure

Blood Cd levels were measured in venous whole-blood samples collected during a baseline visit (2001–2002) conducted according to the standard KoGES protocol. Blood Cd levels (µg/L) were assayed by graphite furnace atomic absorption spectrometry using the SpectrAA-800 Zeeman (VARIAN, Australia). Blood Cd levels were analyzed as both continuous and categorical variables (<0.5 µg/L, 0.5–1.0 µg/L, and >1.0 µg/L). The blood Cd levels in population who were not exposed to Cd are typically lower than 0.5 μg/L, and values of >1.0 μg/L are considered to indicate Cd exposure or increased body burden [10]. The diagnostic reliability and applicability of blood Cd as a measurement of Cd exposure have been described elsewhere [11].

### 2.3. Assessment of Bone Mineral Density and Outcome

The BMD of each participant was determined by quantitative ultrasound (QUS; Omnisense 7000s, SunlightMedical Ltd., Petah Tivka, Israel), which measured the speed of sound (SOS) in meters per second in the distal radius of the non-dominant arm [12]. QUS is an inexpensive, transportable, and ionizing radiation-free approach, and it has been demonstrated to be as effective as dual-energy X-ray absorptiometry measurement of BMD [13]. The QUS of the radius and calcaneum against hip fractures has reliable discriminatory ability [14]. Measurements were performed three times, and the average of the three values was recorded. The QUS results are expressed as absolute values and T-scores by comparing the SOS of the participant with that of reference subjects (young adults): T-score = (SOS of participant—mean SOS of reference subject)/SD of reference subject [15,16].

Two outcomes were determined: (1) the incidence of osteoporosis and (2) the difference in BMD (ΔBMD). First, osteoporosis was defined as a T-score of −2.5 or lower [5]. Second, ΔBMD was calculated by dividing the largest difference in the T-score from the baseline by the corresponding follow-up period.

### 2.4. Confounders

Clinical and biochemical variables which affect exposure, outcome, or both were controlled [1]. Smoking status was categorized as never, former, and current smoker. Alcohol consumption status was classified as never, former, and current drinker. The daily amount of moderate-intensity physical activity (e.g., brisk walking, woodworking, lawn mowing, skiing, swimming, and playing badminton or tennis) was assessed as 0–30, 30–60, and >60 min/day. Treatment histories for rheumatoid arthritis and prior use of systemic glucocorticoids were categorized as yes or no. Height and weight were measured by trained staff members. BMI was calculated as body weight in kilograms divided by height in meters squared. Creatinine level was measured using a HITACHI Auto Analyzer 7600 (Hitachi, Japan).

### 2.5. Statistical Analyses

All statistical tests were performed using R software (version 3.6.3, R Foundation, Vienna, Austria) and GraphPad Prism 9.0 software (GraphPad Software Inc., San Diego, CA, USA). Baseline characteristics were expressed as mean ± standard deviation, and categorical variables were expressed as numbers and percentages. Differences between groups were assessed using independent sample *t*-tests and one-way analysis of variance for continuous variables and chi-square tests for categorical variables. Multivariate Cox regression analysis was performed to assess the effect of Cd exposure and the incidence of osteoporosis. The time of the diagnosis was defined as the date (YYYYMM) of the corresponding survey. For the Cox regression analysis, the follow-up period from baseline (YYYYMM) to the first osteoporotic event (YYYYMM) was calculated at 1-year intervals (range: 2–10 years). After the first osteoporotic event, participants were followed up until they were lost to follow-up. The linear regression analysis was performed to assess the effect of Cd exposure on the ΔBMD. Statistical significance (*p*) was set at <0.05.

## 3. Results

### 3.1. Baseline Characteristics

There were 122 (50.2%) men and 121 (49.8%) women. The mean age of men and women were 57.6 ± 5.1 years and 55.9 ± 6.7 years, respectively. The baseline characteristics by blood Cd concentration (<0.5 μg/L, 0.5–1.0 μg/L, and >1.0 μg/L) of the participants are presented in Table 1. There were significant differences in sex, smoking status, and moderate-intensity physical activity. The prevalence of blood Cd concentrations greater than 1.0 μg/L was higher in women than in men. The current smoker group had a higher prevalence of blood Cd concentration level >1.0 μg/L than the other subgroups of smoking status. Meanwhile, a group with moderate-intensity physical activity for >60 min/day had a lower prevalence of blood Cd concentration level >1.0 μg/L than the other subgroups of physical activity status. The mean age, drinking status, and history of treatment for rheumatoid arthritis were not different among blood Cd concentration groups, and none of the participants used systemic glucocorticoids. The mean values of creatinine and baseline BMD were not statistically different according to blood Cd concentrations.

The personal characteristics of participants by sex are presented in Supplementary Table S1. There was a significant difference between men and women in terms of age, smoking status, drinking status, history of treatment for rheumatoid arthritis, BMI, creatinine, and baseline BMD. The amounts of moderate-intensity physical activity and blood Cd levels were not different between sex.

### 3.2. Associations between Blood Cadmium Level and Osteoporosis and ΔBMD

The average follow-up period was 6.3 ± 3.0 years (range: 2–10 years): 19 (7.8%) and 82 (33.7%) participants were followed up for 2 years and 10 years, respectively. Participants underwent BMD testing an average of three times (range: 2–6), and osteoporosis has occurred in 54 (22.2%; 10 men and 44 women) participants. The individual replicates of T-scores by blood Cd concentration (<0.5 μg/L, 0.5–1.0 μg/L, and >1.0 μg/L) are illustrated in Supplementary Figure S1.

**Figure 1:**
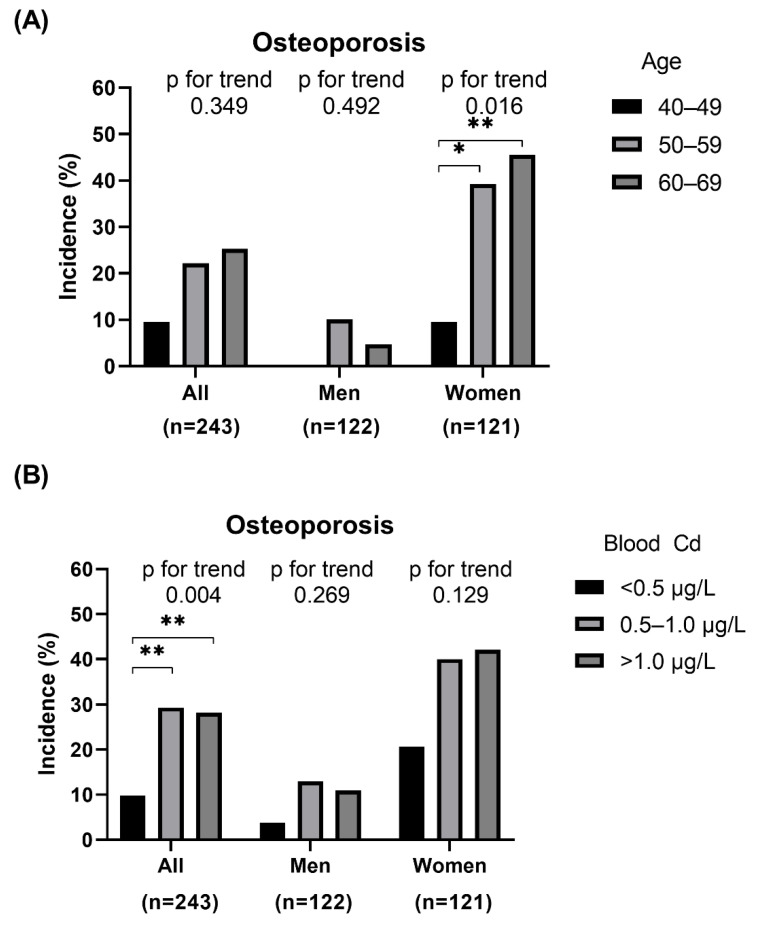
Prevalence of osteoporosis classified by sex, age, and blood cadmium (Cd) level: (**A**) Overall and sex-specific prevalence of osteoporosis according to age, (**B**) Overall and sex-specific prevalence of osteoporosis according to blood Cd level; * *p* < 0.05; ** *p* < 0.01.

The incidence of osteoporosis categorized by sex, age (40–49, 50–59, and 60–69 years), and blood Cd concentration (<0.5 μg/L, 0.5–1.0 μg/L, and >1.0 μg/L) is shown in Figure 1. According to age, in women, the incidence of osteoporosis consistently increased from 9.5% for ages 40–49 years to 45.5% for ages 60–69 years (*p* for trend = 0.016; Figure 1A). Of all participants, the incidence of osteoporosis was significantly different among blood Cd groups (*p* for trend = 0.004); the incidence of osteoporosis was 29.3 and 28.2% in blood Cd groups 0.5–1.0 μg/L and >1.0 μg/L, respectively, which was significantly higher than blood Cd group <0.5 μg/L (9.8%) (both *p* < 0.01). Though the incidence of osteoporosis increased according to blood Cd groups in both men and women, it was not statistically significant (both *p* for trend > 0.05; Figure 1B).

**Figure 2:**
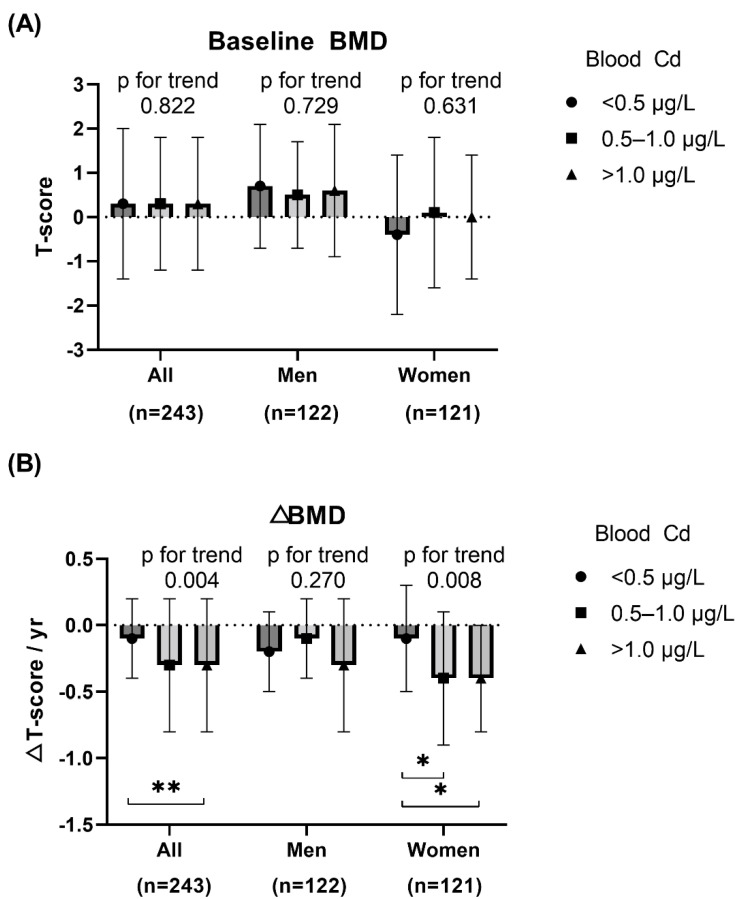
Baseline BMD and ΔBMD of study participants by blood cadmium (Cd) level: (**A**) Overall and sex-specific baseline BMD according to blood Cd level, (**B**) Overall and sex-specific ΔBMD according to blood Cd level; bullet indicates mean, and bar indicates SD. * *p* < 0.05; ** *p* < 0.01.

Baseline BMD and ΔBMD categorized by sex and blood Cd (<0.5 μg/L, 0.5–1.0 μg/L, and >1.0 μg/L) is shown in Figure 2. The baseline BMD did not differ according to blood Cd concentration. However, ΔBMD was significantly lower in groups with blood Cd of 0.5–1.0 μg/L and >1.0 μg/L compared to blood Cd < 0.5 μg/L among all participants (*p* for trend = 0.004), especially in women (*p* for trend = 0.008).

### 3.3. Adjusted Regression Models between Blood Cadmium and Osteoporosis and ΔBMD

Overall and sex-specific cox-regression analysis was performed to determine the effect of blood Cd on the incidence of osteoporosis (Table 2). The regression models were adjusted as follows: Model 1, unadjusted; Model 2, adjusted for age and sex; and Model 3, Model 2 plus smoking status, moderate-intensity physical activity, BMI, creatinine, and baseline BMD. After adjustment, of all participants, blood Cd concentration of >1.0 μg/L (hazard ratio (HR) = 2.67; 95% confidence interval (CI), 1.03–6.91; *p* = 0.043) was associated with the risk for osteoporosis incidence. Particularly in women, blood Cd concentrations of 0.5–1.0 μg/L (HR = 3.8; 95% CI, 1.12–12.84; *p* = 0.032) and >1.0 μg/L (HR = 4.24; 95% CI, 1.25–14.42; *p* = 0.021) were associated with the risk of osteoporosis.

Overall and sex-specific linear regression analyses were performed to determine the effect of blood Cd on the ΔBMD (Table 3). The linear regression models were adjusted the same as the Cox regression models. In the unadjusted model, blood Cd concentrations of 0.5–1.0 ug/L and >1.0 ug/L were negatively associated with ΔBMD in all participants, especially in women. After adjustment, blood Cd concentration of >1.0 μg/L (coefficient = −0.15; 95% CI, −0.28–0.03; *p* = 0.028) was associated with ΔBMD in total participants. In women, the impact of blood Cd on ΔBMD was valid in Model 2, but its impact was attenuated in Model 3.

## 4. Discussion

This study demonstrated that among 122 men aged ≥50 years and 121 post-menopausal women, the incidence of osteoporosis was 22.2% during an average follow-up of 6 years. The incidence of osteoporosis was higher in older and female patients. According to the blood Cd concentration (<0.5 μg/L, 0.5–1.0 μg/L, and >1.0 μg/L), the incident osteoporosis increased, and ΔBMD decreased in sequential order, especially in women. A blood Cd concentration of >1.0 μg/L was a risk factor for incident osteoporosis and decrements in ΔBMD, independent of age, sex, smoking, physical inactivity, BMI, creatinine, and baseline BMD.

In addition to the presence of endocrine disorders (e.g., type 1 diabetes, hyperthyroidism, hypogonadism, or premature menopause), environmental substances should be considered for the cause of secondary osteoporosis. There is emerging evidence that exposure to Cd, lead, phthalates, and perfluoroalkyl substances cause alterations to bone metabolism and lead to osteoporosis [1]. A meta-analysis of 14 studies reported that Cd exposure was associated with a 1.35 times higher risk of osteoporosis, and this risk was higher in older (>65 years) adults [17]. In addition, most studies suggest that the adverse effect of environmental substances on osteoporosis is dominant in post-menopausal women. This might be caused by the synergistic effect of estrogen deficiency and the anti-estrogenic (or anti-androgenic) effects of environmental substances [18].

The potential mechanisms for the Cd effect on bones have been explored. Three months of Cd exposure in female rats via drinking water resulted in an increase in serum calcium, phosphorous, and parathyroid hormone and a reduction in serum vitamin D, osteocalcin, and bone-specific alkaline phosphatase [19]. The Cd treatment of rats induced a reduction in the antioxidant enzyme activity and an increase in malondialdehyde level, resulting in a reduction of viability and proliferation ability of bone marrow mesenchymal stem cells (BMMSCs) [20]. Cd impaired osteogenic differentiation, increased adipogenesis of BMMSCs, and increased cellular senescence through over-activation of the NF-κB signaling pathway [21]. Long-term Cd exposure suppressed P2X7-PI3K-AKT signaling, which inhibited BMMSC osteogenesis and osteoclast differentiation in vitro [22].

In this study, 103 (42.4%) participants showed blood Cd levels higher than 1.0 μg/L, suggesting exposure to Cd [10]. In studies that enrolled participants who were environmentally or occupationally exposed to Cd, there was a dose–response relationship between Cd dose and osteoporosis [23,24], irrespective of renal tubular dysfunction [25,26,27]. The likely source of Cd-related osteoporosis was cigarette smoking in men [28], whereas dietary Cd in women [29,30,31]. In the US general population, the risk for osteopenia and osteoporosis increased with two urinary Cd levels of 1–1.99 μg/g creatinine and ≥2 μg/g creatinine, respectively, independent of age, sex, ethnicity, BMI, calcium intake, and physical inactivity [32]. In addition, even the lower exposure to Cd (urine Cd >0.5 μg/g creatinine) increased the risk of osteoporosis by 2.2 times in men [33] and 1.43 times in women [29]. Our study also suggested that after adjusting for the possible cause of Cd exposure and osteoporosis, blood Cd levels of >1.0 μg/L were associated with a 2.67 times higher risk of osteoporosis, and women were more vulnerable to osteoporosis even at lower doses of Cd levels of 0.5–1.0 μg/L. As there is still controversy over the benchmark dose, further research to specify low-level exposure thresholds in various ethnicities might be beneficial [34].

This study had some limitations. First, the number of participants enrolled was relatively small. Second, there were no data on osteoporotic fracture. Third, the comorbid conditions that can affect osteoporosis (e.g., cancer) were not considered. Fourth, the blood Cd concentration was measured, which reflects short-term exposure rather than long-term accumulation, which urine Cd reflects [35]. However, recent studies have shown the potential role of blood Cd levels as useful biomarkers for osteoporosis [36,37,38,39]. Lastly, this study used QUS and only measured peripheral BMD, which is insufficient to diagnose osteoporosis according to established guidelines [5]. Therefore, larger prospective studies using dual-energy X-ray absorptiometry are warranted.

Despite these limitations, the main strength of this study is that it documented the 10-year impact of Cd exposure on the risk of incident osteoporosis, which is a less explored area of research. In addition, we reported sex-specific effects that were valid after adjusting for important confounders. Lastly, this study is based on a high-quality data source, the KoGES.

## 5. Conclusions

In conclusion, exposure to Cd prospectively increases the incidence of osteoporosis and induces decrements of ΔBMD, especially in Korean women.

## Figures and Tables

**Table 1:** Characteristics of participants by blood cadmium concentration.

	BCd			*p*-Value
	<0.5 µg/L	0.50–1.0 µg/L	>1.0 µg/L	
N (%)	82	58	103	
Sex, *n* (%)				
Male	53 (64.6)	23 (39.7)	46 (44.7)	0.005
Female	29 (35.4)	35 (60.3)	57 (55.3)	
Age, years, *n* (%)				
40–49	5 (6.1)	5 (8.6)	11 (10.7)	0.175
50–59	43 (52.4)	28 (48.3)	64 (62.1)	
60–69	34 (41.5)	25 (43.1)	28 (27.2)	
Age, mean ± SD	57.4 ± 6.1	57.2 ± 6.1	55.9 ± 5.8	0.203
Smoking, *n* (%)				
Never	46 (56.1)	39 (69.6)	61 (60.4)	0.016
Former	20 (24.4)	6 (10.7)	9 (8.9)	
Ever	16 (19.5)	11 (19.6)	31 (30.7)	
Drinking, *n* (%)				
Never	32 (39.5)	30 (52.6)	47 (46.1)	0.27
Former	12 (14.8)	3 (5.3)	8 (7.8)	
Current	37 (45.7)	24 (42.1)	47 (46.1)	
Moderate-intensity physical activity, *n* (%)				
0–30 min/day	42 (54.5)	29 (52.7)	65 (69.1)	0.018
30–60 min/day	20 (26.0)	7 (12.7)	12 (12.8)	
>60 min/day	15 (19.5)	19 (34.5)	17 (18.1)	
Treatment of rheumatoid arthritis, *n* (%)				
No	57 (95.0)	34 (94.4)	50 (89.3)	0.499
Yes	3 (5.0)	2 (5.6)	6 (10.7)	
Prior use of systemic glucocorticoids, *n* (%)				
No	0 (0)	0 (0)	0 (0)	NA
Yes	0 (0)	0 (0)	0 (0)	
BMI, kg/m^2^	24.4 ± 3.4	24.6 ± 3.4	24.6 ± 3.3	0.963
Cre, mg/dL	0.9 ± 0.2	0.8 ± 0.2	0.8 ± 0.2	0.201
Baseline BMD, T-score	0.3 ± 1.7	0.3 ± 1.5	0.3 ± 1.5	0.950

BMD—bone mineral density; Cre—creatinine; SD—standard deviation; BCd—blood cadmium; BMI—body mass index; NA—not available.

**Table 2:** Cox regression analysis of the association between blood cadmium levels and the incidence of osteoporosis.

	Model 1 HR (95% CI)	Model 2 HR (95% CI)	Model 3 HR (95% CI)
Total			
Blood Cd: ref. < 0.5 µg/L			
0.5–1.0 µg/L	2.46 (1.06, 5.72) *	1.69 (0.72, 3.95)	2.33 (0.87, 6.2)
>1.0 µg/L	2.34 (1.07, 5.15) *	1.9 (0.87, 4.19)	2.67 (1.03, 6.91) *
Men			
Blood Cd: ref. < 0.5 µg/L			
0.5–1.0 µg/L	2.27 (0.37, 13.88)	2.27 (0.37, 13.77)	0.84 (0.07, 9.71)
>1.0 µg/L	2.08 (0.4, 10.93)	2.14 (0.41, 11.17)	1.53 (0.21, 11.15)
Women			
Blood Cd: ref. < 0.5 µg/L			
0.5–1.0 µg/L	1.86 (0.71, 4.84)	1.59 (0.61, 4.15)	3.8 (1.12, 12.84) *
>1.0 µg/L	1.82 (0.74, 4.45)	1.95 (0.79, 4.79)	4.24 (1.25, 14.42) *

HR—hazard ratio; CI—confidence interval; Ref—reference; Cd—cadmium. Model 1: unadjusted. Model 2: adjusted for age and sex. Model 3: adjusted for Model 2 plus smoking status, moderate-intensity physical activity, BMI, creatinine level, and baseline BMD. * *p* < 0.05.

**Table 3:** Linear regression analysis of the association between blood cadmium levels and the ΔBMD (/per year).

	Model 1 Coefficient (95% CI)	Model 2 Coefficient (95% CI)	Model 3 Coefficient (95% CI)
Total			
Blood Cd: ref. <0.5 µg/L			
0.5–1.0 µg/L	−0.14 (−0.28, 0) *	−0.12 (−0.26, 0.02)	−0.08 (−0.22, 0.06)
>1.0 µg/L	−0.18 (−0.3, −0.06) **	−0.17 (−0.3, −0.05) **	−0.15 (−0.28, −0.03) *
Men			
Blood Cd: ref. <0.5 µg/L			
0.5–1.0 µg/L	0.02 (−0.16, 0.2)	0.02 (−0.16, 0.2)	0.08 (−0.1, 0.25)
>1.0 µg/L	−0.12 (−0.26, 0.03)	−0.12 (−0.26, 0.03)	−0.08 (−0.22, 0.07)
Women			
Blood Cd: ref. <0.5 µg/L			
0.5–1.0 µg/L	−0.28 (−0.5, −0.06) *	−0.27 (−0.49, −0.05) *	−0.13 (−0.36, 0.1)
>1.0 μg/L	−0.26 (−0.46, −0.06) *	−0.26 (−0.46, −0.06) *	−0.2 (−0.41, 0.01)

BMD—bone mineral density; CI—confidence interval; Ref—reference; Cd—cadmium. Model 1: unadjusted. Model 2: adjusted for age and sex. Model 3: adjusted for Model 2 plus smoking status, moderate-intensity physical activity, BMI, creatinine level, and baseline BMD. * *p* < 0.05; ** *p* < 0.01.

## Data Availability

Restrictions apply to the availability of these data. Data were obtained from the Korea Disease Control and Prevention Agency and are available with the permission of the Korea Disease Control and Prevention Agency.

## Supplementary Materials

The following supporting information can be downloaded at: https://www.mdpi.com/article/10.3390/jcm11102899/s1, Figure S1: Individual replicates of bone mineral density by blood Cadmium concentration; Table S1: Personal characteristics of participants by sex.
