# Supplementary material for: Long-Term Sex-Specific Effects of Cadmium Exposure on Osteoporosis and Bone Density: A 10-Year Community-Based Cohort Study
Source: J Clin Med. 2022 May 20;11(10):2899. doi: 10.3390/jcm11102899 (PMC9145052; doi:10.3390/jcm11102899)
Supplement: Supplementary file 1 [file jcm-11-02899-s001.zip › jcm-1702671-supplementary.pdf]

Table S1. Personal characteristics of participants by sex.

	Men	Women	p-value
N (%)	122	121	
Age, n(%)			
40-49	0 (0)	21 (17.4)	<0.001
50-59	79 (64.8)	56 (46.3)	
60-69	43 (35.2)	44 (36.4)	
Age, Mean ± SD	57.6 ± 5.1	55.9 ± 6.7	0.023
Smoking, n(%)			
Never	34 (27.9)	112 (95.7)	<0.001
Former	35 (28.7)	0 (0.0)	
Current	53 (43.4)	5 (4.3)	
Drinking, n(%)			
Never	25 (20.5)	84 (71.2)	<0.001
Former	20 (16.4)	3 (2.5)	
Current	77 (63.1)	31 (26.3)	
Moderate intensity physical activity, n(%)			
0-30min/day	71 (62.8)	65 (57.5)	0.353
30-60min/day	21 (18.6)	18 (15.9)	
>60min/day	21 (18.6)	30 (26.5)	
Treatment of rheumatoid arthritis, n(%)			
No	75 (98.7)	66 (86.8)	0.012
Yes	1 (1.3)	10 (13.2)	
Prior use of systemic glucocorticoids, n(%)			
No	0 (0)	0 (0)	NA
Yes	0 (0)	0 (0)	
BMI, kg/m^2	23.7 ± 3.0	25.4 ± 3.4	<0.001
Cre, mg/dL	0.9 ± 0.2	0.8 ± 0.1	<0.001
Cadmium, μg/L	1.0 ± 1.3	1.3 ± 1.1	0.115
Baseline BMD, T-score	0.7 ± 1.4	-0.1 ± 1.6	<0.001

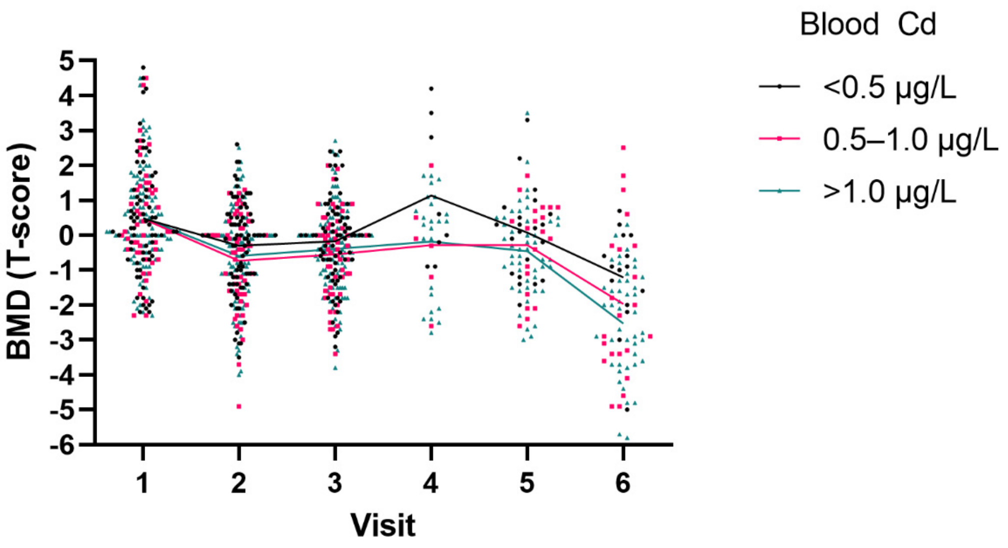

Figure S1. Individual replicates of bone mineral density by blood Cadmium concentration. Line indicates mean value of T-scores for each visit by blood cadmium. Visit 1, 2001-2002; visit 2, 2003-2004; visit 3, 2005-2006; visit 4, 2007-2008; visit 5, 2009-2010; visit 6, 2011-2012.
